# Defect Imaging Enhancement through Optimized Shape Factors of the RAPID Algorithm Based on Guided Wave Beam Pattern Analysis

**DOI:** 10.3390/s21124029

**Published:** 2021-06-11

**Authors:** Yonghee Lee, Younho Cho

**Affiliations:** 1 Graduate School of Mechanical System Design, Pusan National University, Busan 46241, Korea; leedragon83@pusan.ac.kr; 2 School of Mechanical Engineering, Pusan National University, Busan 46241, Korea

**Keywords:** tomography, image modification, mode verification, guided wave, beam pattern analysis, signal processing

## Abstract

In this study, a modified imaging algorithm was implemented to improve the imaging accuracy for defects located on a structure. Based on analysis of the Lamb wave mode, a guided ultrasonic wave inspection technique was applied, which was able to illustrate images of defects in a 6 mm steel plate simulating containment liner plate (CLP) in nuclear power plants. The dominant Lamb wave mode was determined through short-time Fourier transform waveform analysis and imaging verification. Following tomography verification, limitations of the antisymmetric mode in the thick steel plate were identified. In addition, a modified shape factor, based on the energy distribution factor according to the beam pattern and beam width, was suggested for field applications and improved imaging accuracy. Results of the analysis revealed a beam skewing phenomenon for the Lamb wave mode. In the case of S0 2.7 MHz·mm, skewing as well as distortion effects are not observed in the experiment, while the S0 modes at 2.64 and 2.74 MHz·mm show either of them. Considering skewing width, the size of the shape function was modified. Application of the modified shape function allows us to obtain more accurate image to actual defect shape.

## 1. Introduction

From the viewpoint of mechanical and structural engineering, effective assessment of huge facilities and instruments has already been conducted to verify safety factors. There are two parts of an integrity assessment: a destructive test, which relies on fracture mechanics, and a nondestructive test, without any significant external impact. To understand these technologies, a basic grasp of the theory of mechanics and physical phenomena is required. To consider field workers unfamiliar with the technical background of nondestructive evaluation (NDE) or test (NDT), the monitoring technology needs to focus on an intuitive, easy understanding. A structural health monitoring (SHM) system is an excellent means to determine the condition of a structure. Many essential techniques are required to setup real-time health monitoring systems, such as visualization, sensor networking, and signal processing. Most importantly, high resolutions and accurate tomographic techniques are the key tools for operating health monitoring systems. In this study, ultrasonic waves, especially guided waves, were utilized for systemizing an SHM system. Because of several environmental limitations, such as radioactive surroundings, it was difficult to perform the radioactive test or eddy current test to set up the SHM system. The alternative physical phenomenon employed was ultrasonic wave assessment using guided waves; ultrasonic guided waves exhibit high sensitivity and accessibility along with a sufficient detection range. The physical phenomena and fundamental theory of guided waves have been discussed in many well-known studies and texts [1,2,3].

Many studies have been conducted on visualization techniques, including tomography. The development of the tomography technique has led to the concept of X-ray image processing [4]. Owing to the characteristic of intuitional advantage, image processing technologies are developed primarily in the medical field. The computed tomography method has facilitated significant progress in medical diagnosis and detectability of pathogenesis. Based on these ideas, ultrasonic diagnosis techniques are widely applied in high-risk industrial fields, such as nuclear power plants, bridge construction, and aircraft. To evaluate the integrity of a plate-like structure, a guided wave inspection technique was developed based on the Lamb wave synthetic aperture focusing technique (L-SAFT), which is a wave synthetic aperture focusing technique (SAFT) [5]. The conventional SHM technique based on the ultrasonic guided wave method is a sensor-insertion type approach on the body, which affects the strength and durability of an airplane and the repairs the diagnosis system. To enhance the operating sensor-based diagnosis system for airplane body conditions, a contact-based SHM algorithm was developed using the guided wave reconstruction algorithm for the probabilistic inspection of damage (RAPID) technique [6]. In the case of SHM in a plate, the omnidirectional transducer method was used to mimic the condition of multiple unidirectional transducers. Considering the guided wave behavior, the plate’s integrity was successfully illustrated through defect imaging [7]. A Lamb wave based cross-hole visualization approach was also investigated. For pipe structure comparison, the meridional ultrasound tomography and helical ultrasonic tomography techniques were verified for reliability and complementary effects [8]. Another significant study was undertaken using the Lamb wave. As the thickness of the plate changed, the time domain of time of flight tended to be the key factor in visualization of the wall-thinning status [9]. A study of the implementation of tomography resolution was undertaken to consider the diffraction effect of guided waves [10]. A number of case studies and comparison reviews for various tomographic approaches were considered. Three algorithms—the time-of-arrival imaging method, time-difference-of-arrival imaging method, and RAPID technique—were compared, and the pros and cons of each visualization technique were analyzed [11]. In this study, the RAPID algorithm played a significant role in the visualization results. Many researchers have tried to focus on establishing an SHM system and providing a solution for inaccessible defects in structures. For applications of the RAPID algorithm, the variable shape factor was investigated by considering the location and shape of the defects (circular and quadrilateral shapes) [12]. The variable shape factor approach shows a remarkable enhancement of reconstruction of defect images. In the nuclear power plant industry, the containment liner plate assessment technique is of great importance for the prevention of de-bonding and wall-thinning failure. The evaluation of the CLP condition was researched based on the guided wave method and RAPID algorithm. The wall-thinning condition was successfully indicated by the technology [13]. Furthermore, a visualization image with enhanced resolution and precision was developed by applying the full-wave-inversion algorithm [14,15]. To enhance the accuracy of defect reconstruction images, alternative ways are developed. For inspection of an oil–water biphasic medium, regarded as the tank storage, a modified ray-tracing technology is researched [16]. Additionally, the ray tomography concept is used to evaluate temperature–robust damage in the structure [17]. In the viewpoint of scattering and propagating, advanced ways are investigated. The quality of guided wave tomography is developed by analysis of scattering patterns [18]. In the same manner, laminar damages are treated by using near-field imaging approaches [19]. A noncontact-based image reconstruction technique, application of an electromagnetic transducer, is used for plate-like structures [20,21,22]. Not only plate-like structures, but also the pipe and various geometrical conditions are investigated. From the analysis of the attenuation effect of the guided wave, the filling distribution and height levels are investigated for insulated pressure vessel structure [23]. In the case of a pipe, synthetic verification is investigated using the wavefield cross-correlation technique [24]. A long-term monitoring system is conducted to assess the pipe bending case by considering environmental conditions [25]. In addition, a microdamage reconstruction technique is developed. From the analysis of frequency level, evaluating third-order elastic constant, microdefects are illustrated by the application of the RAPID algorithm [26]. Another approach, analysis of pulse–echo modes, is also investigated depending on the Westervelt equation [27]. In the case of diagnosis of multiple-dynamics equipment, electric impact drills, thermal imaging processing is researched [28]. The technique is based on a thermal image process, known as binarized common areas of image difference, which are processed by an artificial neural network and K-nearest neighbor. Recently, the image processing algorithm is remedied by the application of the fuzzy process. In the stage of illustration and displaying defect images, fuzzy processing is expected to elevate the quality of tomographic images [29,30].

In recent years, much research has aimed at developing the resolution of the imaging process and the applicability of SHM and real-time based assessment systems. Despite these engineering enhancements, in the case of application of the RAPID algorithm, an imaging revision technique is required to illustrate defects with good reconstruction. The variable shape factor is demonstrated effectively in the RAPID algorithm. However, in field conditions, the technique has limitations. Advanced RAPID tomographic techniques are required to improve the resolution and accuracy of images with respect to the operating surroundings. 

To explain this effectively, abbreviations used are short-time Fourier transforming (STFT), nondestructive evaluation (NDE), nondestructive test (NDT), structural health monitoring (SHM), radioactive test (RT), eddy current test (ECT), computed tomography (CT), Lamb wave synthetic aperture focusing technique (L-SAFT), reconstruction algorithm for probabilistic inspection of damage (RAPID), meridional ultrasound tomography (MUT), helical ultrasonic tomography (HUT), time of flight (TOF), and signal difference coefficient (SDC).

Note that wall-thinning defects on the plate are significantly high-risk and complex problems in the industrial field. A quantitative and reliable revision technique of the RAPID algorithm, based on guided wave SHM, is required to enhance the precision. The present study focused on verifying a proper guided mode and revised shape factor technique by considering experimental and simulated approaches with respect to the tendency of wave propagation and image illustration. First, a simple real-case model was proposed to explain the relationship between the appropriate guided wave modes and the wall-thinning damage states of plates, based on analysis of wave propagation. Next, from a practical viewpoint, guided waves, such as Lamb waves, were generated to verify the appropriate mode using both the finite element and experimental methods. Finally, by analyzing the beam pattern and energy level, the modified shape factor was applied to the reconstruction of images.

## 2. Theoretical Fundamentals

### 2.1. Lamb Wave Mode Verification

**Figure 1:**
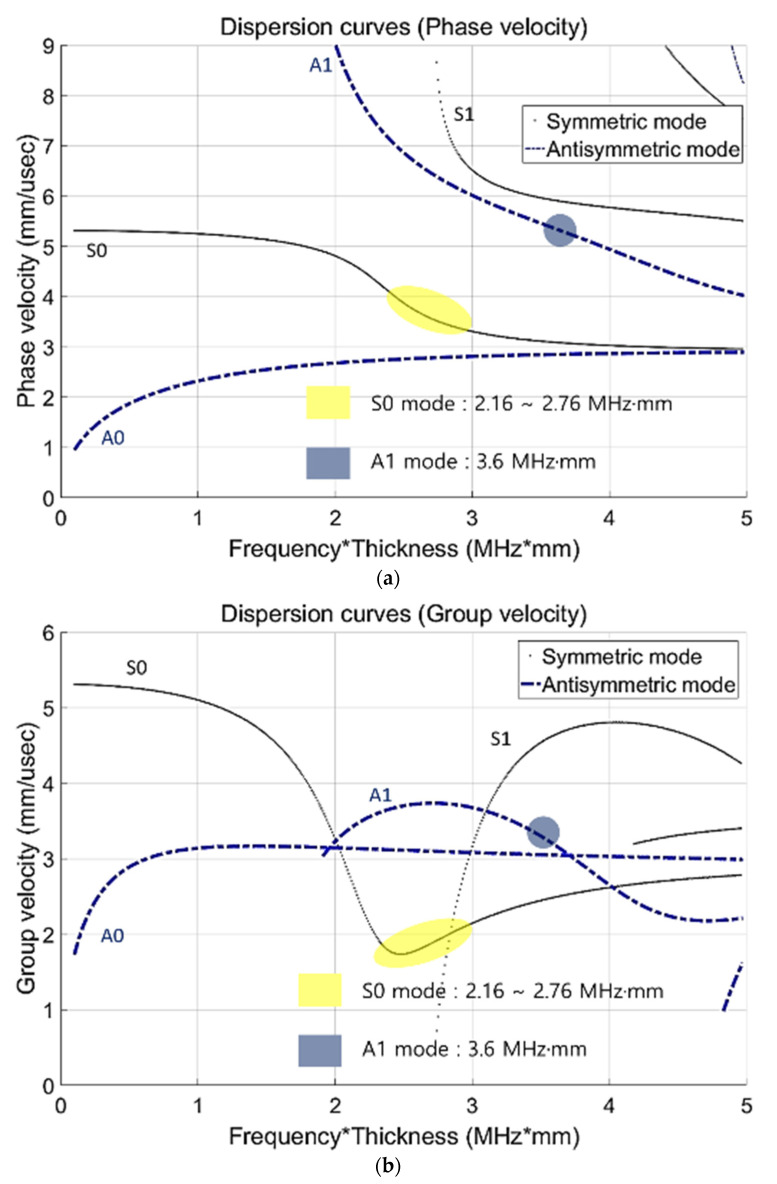
Lamb wave dispersion curves: (**a**) phase velocity and (**b**) group velocity.

In Section 1, many recent studies on Lamb wave physical phenomena are introduced. Lamb wave propagation presents problems associated with wave fluctuation and speed in a homogeneous isotropic material. In terms of the vibrating motion, two wave modes are indicated in the Lamb wave case: a symmetric mode and an antisymmetric mode. The wave mode and speed are defined by velocity of the bulk wave, wave number, and frequency. Most theoretical equations are explained by Rose et al., Cawley et al., and Viktorov et al. [1,2,3]. In this study, the generable frequency range was 300–600 kHz. A higher frequency range (over 1 MHz) wave signal was not suitable for evaluating the assessment of thick plates, owing to insufficient energy maintenance. To begin the experimental investigation, the most crucial step was mode selection using dispersion curve analysis. The dispersion curve was drawn using the bisection [1]. Considering excitation frequency, S0 wave mode was assumed to satisfy Lamb wave modes, based on previous research [13]. The dispersion curve diagram, based on eigenvalue analysis, is illustrated in Figure 1.

The Lamb wave mode needs to satisfy several conditions, including main mode dominance, sufficient propagation distance, and reliability of generation. Considering all terms of these conditions, with a preponderant reliability and dominance mode, S0 and A1 are the serviceable Lamb wave modes. In this study, the symmetric mode S0 (2.16 to 2.76 MHz·mm) was applied to visualize defects. In addition, for comparison of symmetric and antisymmetric transverse wave dominance modes, mode A1 (3.6 MHz·mm) was applied. Based on this comparison, the appropriate mode was chosen, considering the wave characteristics and visualization enhancement.

### 2.2. Basic Principle of the RAPID Algorithm

The central focus of this study was the development of a revised shape function that can evaluate defects with respect to beam skewing and energy distribution. To improve the detection ability, many transducers needed to be setup in high-risk areas of the structure. However, because of inaccessibility and a limited number of transducers, an alternative method that considered the surroundings was required. To overcome these challenges, a revised RAPID algorithm was utilized as an alternative means of examining the structure. This algorithm is based on a signal difference coefficient (SDC), in accordance with its compatibility with embedded sensor applications. The principle of SDC, as expressed in Equation (1), compares the damage and safety statuses associated with the cross-correlation function.

In Equation (1), the time variable $t_{0}$ represents the direct arrival time of each transducer pair; $\mu_{x}$ and $\mu_{y}$ are the means of datasets *X* and *Y*, respectively. *X* is the data in the first step which is propagating on the non-defect zone and the beginning stage of structure and *Y* is the data set acquired after a specified operating time period [6,8,9,12,13,18,19,26]. If the signals are identical to the initial state, the SDC is to be zero, and whether they are completely out of phase, the SDC calculated a maximum value of one. After the SDC values for all transducer sets are analyzed, the next step of the RAPID process is the reconstruction of the image.

(1) $${\mathrm{SDC}}_{ij}=1-\left|\frac{\int_{t_{0}}^{t_{0}+\Delta T}\left[x_{TR}\left(t\right)-\mu_{x}\right]\left[y_{TR}\left(t\right)-\mu_{y}\right]dt}{\sqrt{\int_{t_{0}}^{t_{0}+\Delta T}{\left[x_{TR}\left(t\right)-\mu_{x}\right]}^{2}dt\int_{t_{0}}^{t_{0}+\Delta T}{\left[y_{TR}\left(t\right)-\mu_{y}\right]}^{2}dt}}\right|.$$

A tomographic image is illustrated by distributing each signal difference value in an elliptical shape. A shape coefficient *β* is defined to adjust the size of the ellipse, which depends on the beam pattern and defect size. Note that the shape parameter *β* is considered to be the shape coefficient that controls the size of the elliptical distribution:

(2) $$s\left(x,y,x_{T},y_{T},x_{R},y_{R}\right)=\frac{\beta -R\left(x,y,x_{T},y_{T},x_{R},y_{R}\right)}{1-\beta }\;\beta >R\left(x,y,x_{T},y_{T},x_{R},y_{R}\right)\;s\left(x,y,x_{T},y_{T},x_{R},y_{R}\right)=0,$$

where $R\left(x,y,x_{T},y_{T},x_{R},y_{R}\right)$ is the ratio of the sum of distances of point $\left(x,y\right)$ from the pulsar $\left(x_{T},y_{T}\right)$ and receiver $\left(x_{R,}y_{R}\right)$ and the distance between the pulsar and receiver; it is mathematically stated as:

(3) $$R\left(x,y,x_{T},y_{T},x_{R},y_{R}\right)=\frac{\sqrt{{\left(x-x_{T}\right)}^{2}+{\left(y-y_{T}\right)}^{2}}+\sqrt{{\left(x-x_{R}\right)}^{2}+{\left(y-y_{R}\right)}^{2}}}{\sqrt{{\left(x_{T}-x_{R}\right)}^{2}+{\left(y_{T}-y_{R}\right)}^{2}}}$$

**Figure 2:**
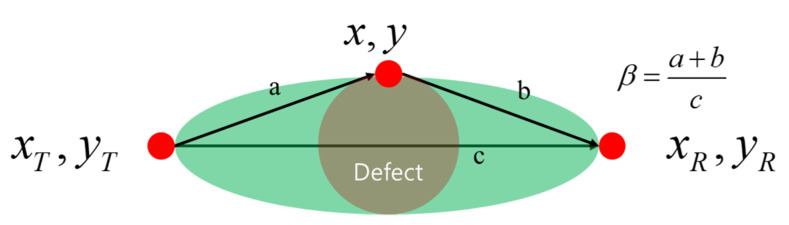
Fundamentals of the shape function.

Figure 2 depicts the fundamentals of the shape factor, as expressed in Equation (2). Herein, *c* is the length of the major axis, and *a* and *b* represent the lengths between the end points of the major axis and the farthest position of the radius of the defect.

Finally, the tomographic image amplitude at each pixel is calculated as the linear summation of the location probabilities for each transmitter and receiver $s\left(x,y,x_{T},y_{T},x_{R},y_{R}\right)$ pair; the total number of transmitter–receiver pairs is denoted by M. The tomographic image amplitude is calculated using the equation:

(4) $$P\left(x,y\right)=\overset{M}{\underset{k=1}{\sum }}p_{k}\left(x,y\right)=\overset{M}{\underset{k=1}{\sum }}\frac{SDC}{\beta -1}\left\{\beta -R(x,y,x_{T},y_{T},x_{R},y_{R}\right\},1\leq R\leq \beta$$

## 3. Method and Experimental Setup

### 3.1. Total Process of Modifying the Shape Function

**Figure 3:**
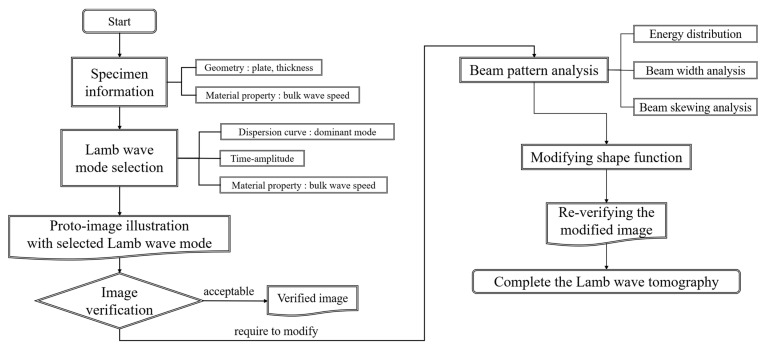
Overall processing of the shape function modification by using the RAPID algorithm.

The modification concept is structured by considering the proper wave mode selection, evaluation of the quality of images, and sound beam pattern analysis. In beam pattern analysis, the wave energy, distributed on the specimen, is the most crucial factor in figuring out the beam path and beam-skewing. The first step is the verification of wave modes. The dominance of mode and the intensity of wave signal are kernel items to consider. The second step is illustrating the image by application of the RAPID algorithm. The reliability of defect images is discussed fully considering distortion effects. In the case of distorted images, the beam pattern analysis is conducted by considering energy distribution, beam width analysis, and beam skewing analysis. The modified shape function is calculated by the rules of the proposed beam pattern analysis and modification technique. And the last step is re-verification. The results of beam pattern nature present the basic information of modifying shape function. The total process of modifying the shape function is explained as shown in Figure 3.

### 3.2. Specimen

**Figure 4:**
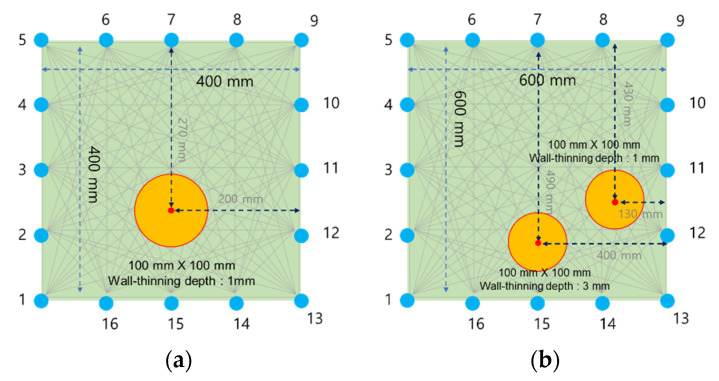
Specimen information: (**a**) single defect and (**b**) multiple defects.

**Figure 5:**
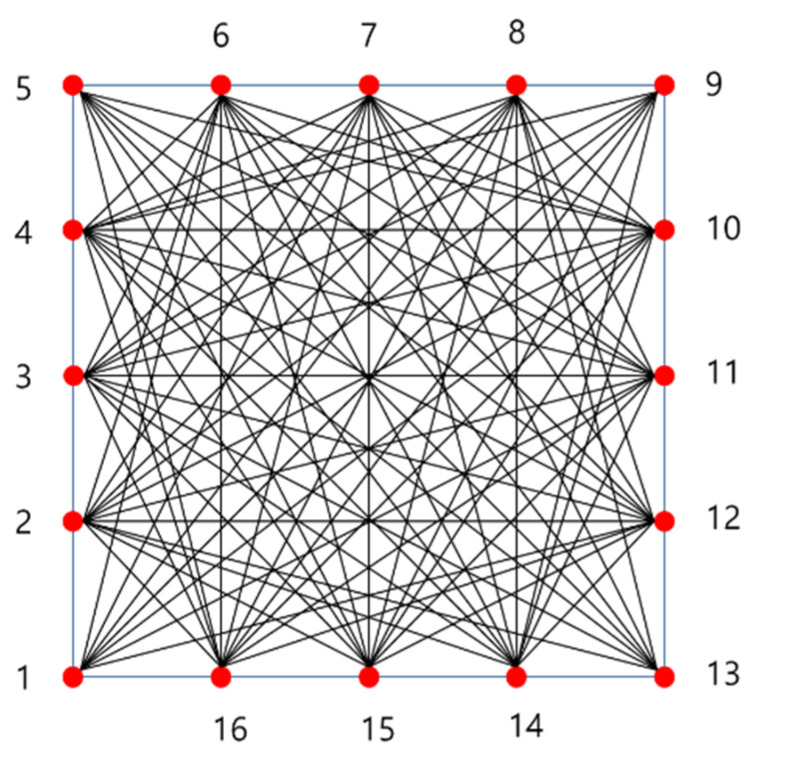
Propagation network for the tomographic image.

The aim of this study was to verify an appropriate mode verification and determine a revised shape factor for defects. To determine the appropriate Lamb wave modes, a relatively bulkier plate was used for the study specimen. For a thickness less than 3 mm, mode identification requires more sophisticated signal analysis. To reduce the disturbance or instrumental error, a low frequency-based wave signal is preferred. In contrast, propagating distance and wave signal attenuation effects are likely to occur in the case of a thicker plate. As per the discussion about the physical meaning of Lamb waves in Section 2, antisymmetric modes are significantly affected by the transverse wave, which is related to the bending effect. Antisymmetric Lamb wave modes are sufficiently excited in the bulky plate case. Recent studies using a treated containment liner plate indicated that a plate with thickness 6 mm is acceptable for Lamb wave propagation [15,16,17]. The specimen information is shown in Figure 4. The carbon–steel plate was composed of SA516 GR 60 material, and its thickness was 6 mm. The dimensions of the plate (width and height) were 400 × 400 mm and 600 × 600 mm with a maximum propagation distance in the range of 141–848 mm. To collect sufficient wave signals, the transducer arrays were located as shown in Figure 5. To address fundamental SHM application concerns, robotics or built-in sensors should be considered. Accordingly, in light of the attenuation of the Lamb wave and the illustration of a maximum-sized tomography, a total of 16 transducer sets were deployed, and 160 wave signals were treated by each local section. At the end of these processes, reconstructed images were obtained through a combination of cross-correlated wave amplitudes.

### 3.3. Experimental Setup

**Figure 6:**
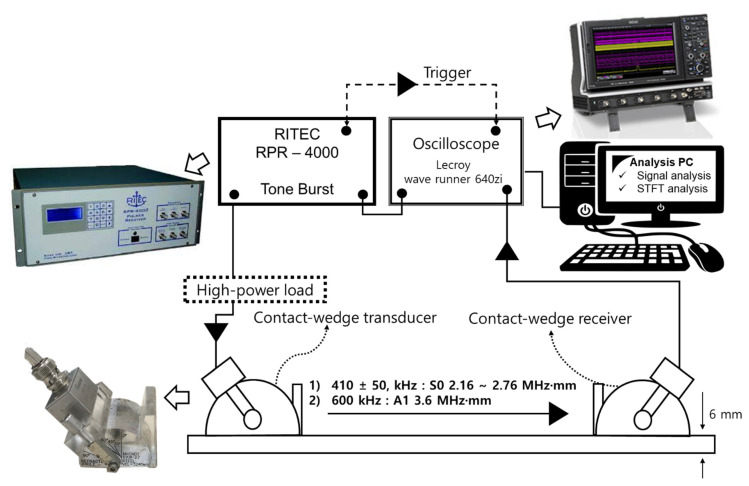
Experimental setup for generating PZT-based Lamb waves.

The experimental setup used to generate a Lamb wave signal with sufficient energy is shown in Figure 6. A high-power tone burst instrument was connected to an oscilloscope and a laptop computer for data analysis. The tone burst instrument is enough to generate the sinusoidal signal that is windowed by the Hann window function. The oscilloscope is capable of analyzing the signal at its maximum sampling rate of 40 GS/s, which can suitably acquire the signal.

To acquire the Lamb wave signal, a piezoelectric transducer (PZT) was applied to the CLP mock-up specimen. The PZT (standard square shape, 12.7 × 25.4 mm^2^) exhibited a center frequency of 400 kHz; its frequency range was 320–580 kHz. Considering availability on wave propagation, a contact-based experiment was performed using an acoustic couplant, instead of a noncontact method. The transducer was manually fixed on the wedge using a pressure of 186 kPa. From an application viewpoint, the noncontact technique was required to excite a wave signal. To determine the best excitation condition, the contact-based transducer array had to determine the feasibility of the most appropriate mode in advance.

The first step was to generate five cycles of sinusoidal signal windowed by the Hann function. Through the high-power load, acting as an attenuator and a capacity, the signal conducted excitation of the PZT and the transducer excited the wave signal and transferred it to the acrylic wedge. Based on Snell’s law, the angle of excitation was determined. Then, the Lamb wave signal was propagating on the waveguide—the CLP. The signal transferred to the transducer and collected in reverse order of the excitation process. The last step was collecting wave signals. All of the signals were saved by the oscilloscope. Following this, the wave signal’s frequencies and amplitudes were analyzed using the laptop computer.

## 4. Wave Mode Analysis

### 4.1. Waveform Analysis

**Figure 7:**
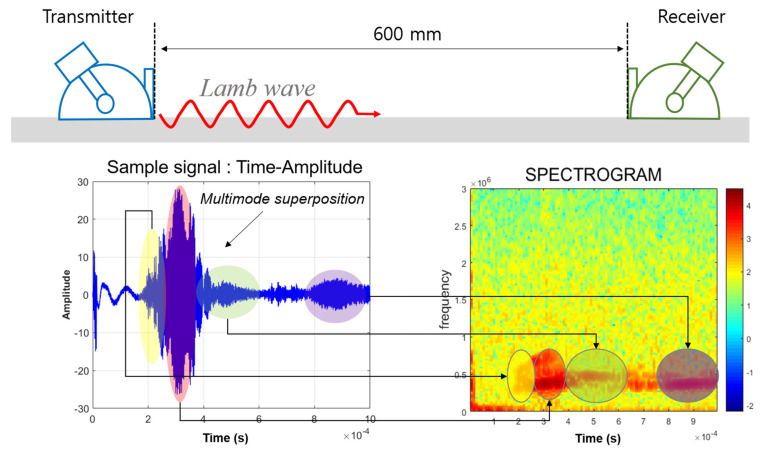
The mode identification process of Lamb waves considering group velocity, frequency, and superposition effect.

**Figure 8:**
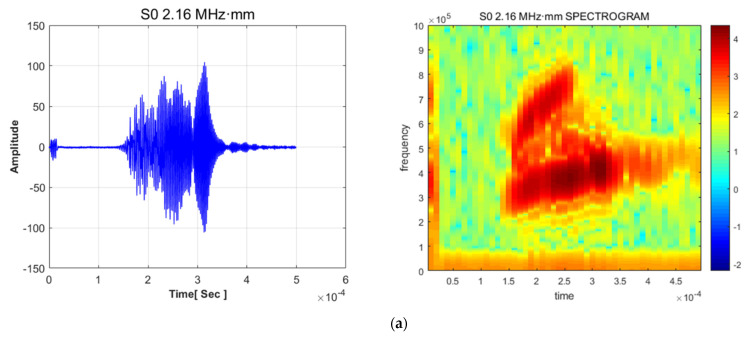

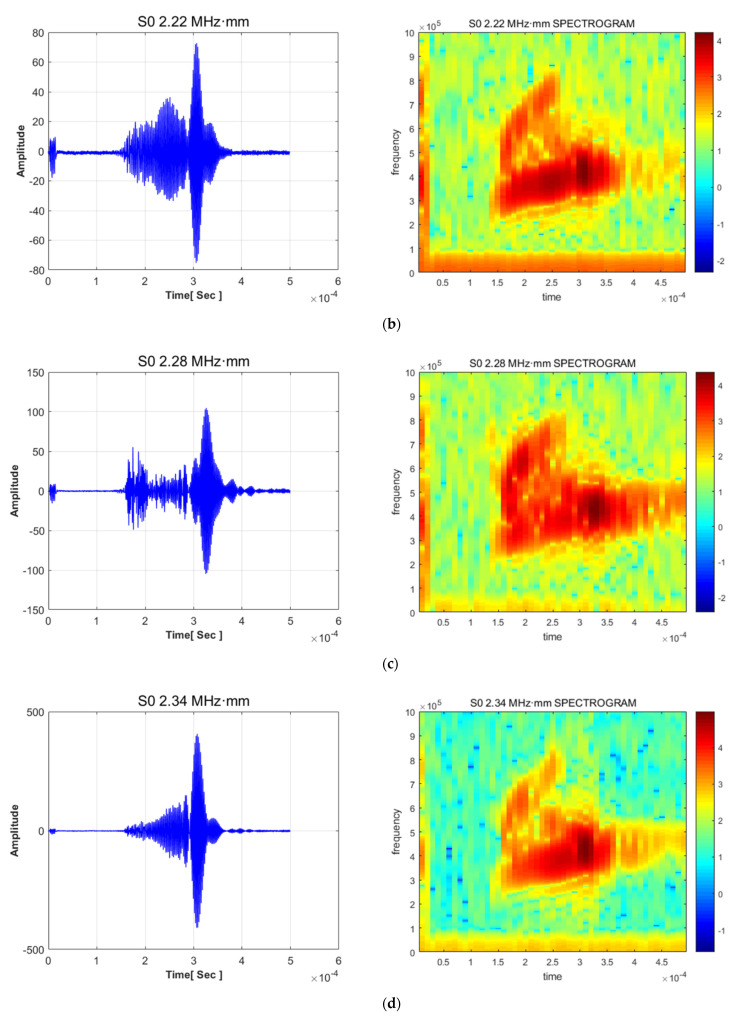

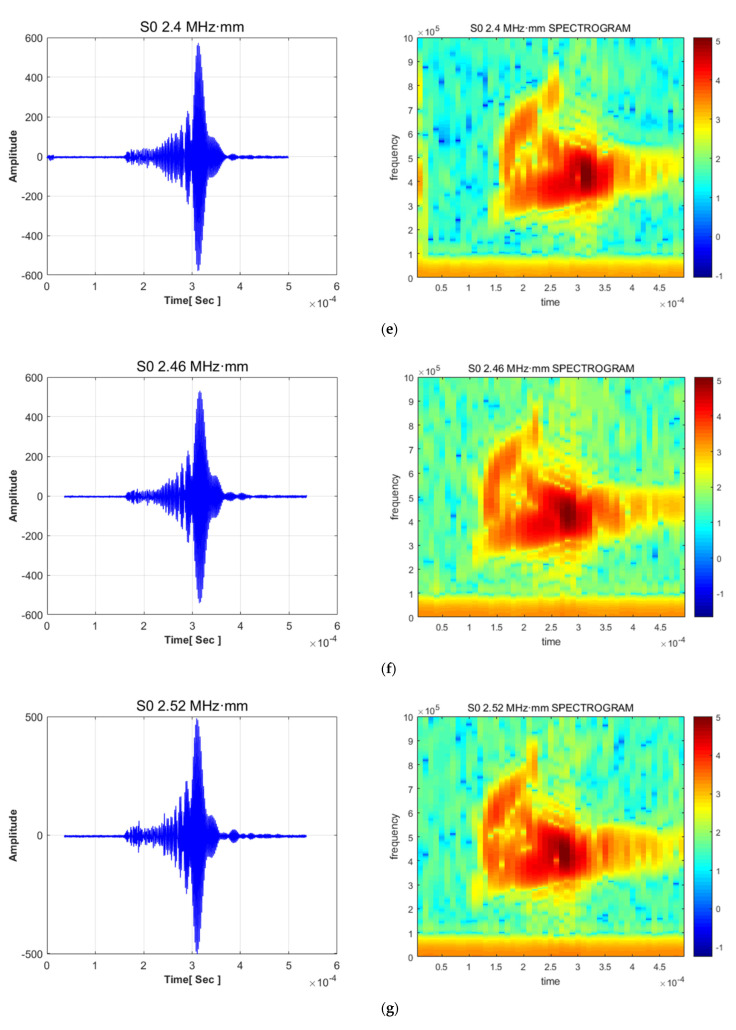

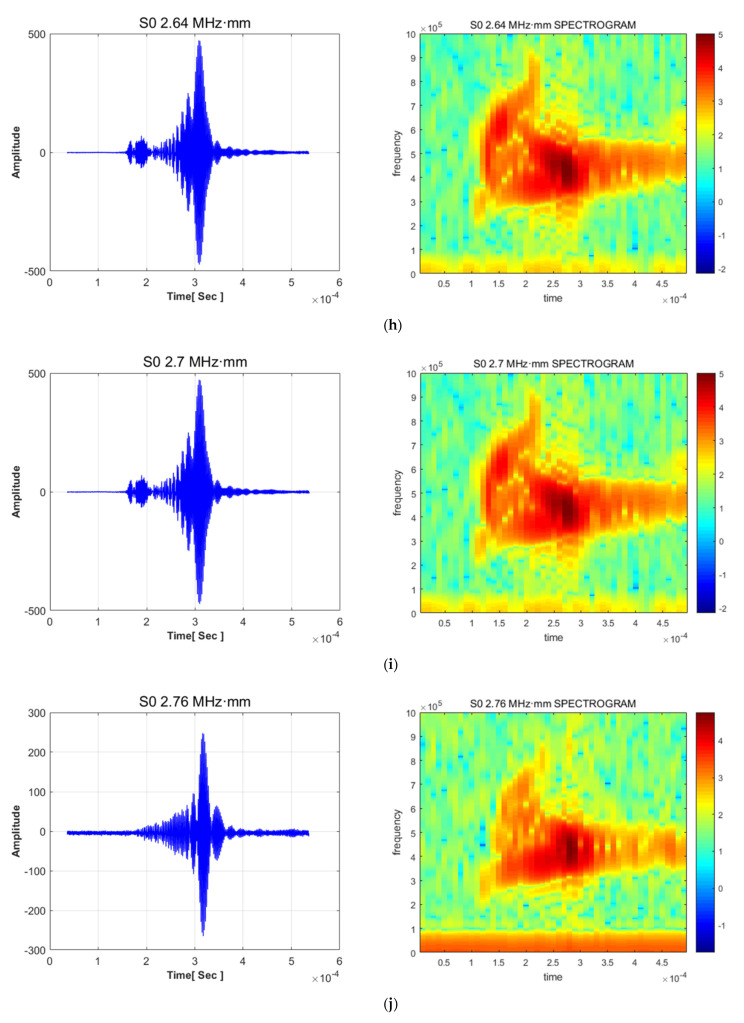
Results of STFT and waveform analyses for symmetric Lamb wave modes: (**a**) S0 2.16 MHz·mm, (**b**) S0 2.22 MHz·mm, (**c**) S0 2.28 MHz·mm, (**d**) S0 2.34 MHz·mm, (**e**) S0 2.40 MHz·mm, (**f**) S0 2.46 MHz·mm, (**g**) S0 2.52 MHz·mm, (**h**) S0 2.64 MHz·mm, (**i**) S0 2.70 MHz·mm, and (**j**) S0 2.76 MHz·mm.

To choose the most prominent mode for illustrating quantitative images, the revised RAPID algorithm with a modified shape factor was employed. The short-time Fourier transform (STFT) method was applied for mode verification; this method determined the frequency and wave speed of the signal. Analysis of the waveforms and STFT revealed that for frequencies ranging from 2.16 to 2.52 MHz in the symmetric mode case, wave dominance was rarely indicated with the mode superposition effect. The procedure of signal analysis was investigated considering group velocity and mode dominance. The dominance of mode is a significant factor when applying the Lamb wave inspection technique. Each mode’s sensitivity is different, which can affect evaluation defects. The basic concept of the process is illustrated in Figure 7. For waveform analysis, multiple wave signals were simultaneously generated in the plate. Results of the STFT and waveform analyses are demonstrated in Figure 8. Waveform analysis of the 2.64–2.76 MHz range indicates that the waveform and frequency show good dominance of excitation. In addition, noise and disturbances rarely appear in the spectrograph.

According to the theory of the RAPID algorithm described in Section 2, the SDC is defined by the difference in amplitude for the safety and failure conditions. Therefore, we illustrated the defect using nondominant wave modes. To examine mode identification, group velocity analysis was conducted by means of main time–amplitude signal analysis. The group velocities for each mode are listed in Table 1. Comparisons between the experimental and theoretical velocities indicate that most of the wave modes are excited without any conspicuous disturbance signal.

### 4.2. Tomographic Effect Analysis

**Figure 9:**
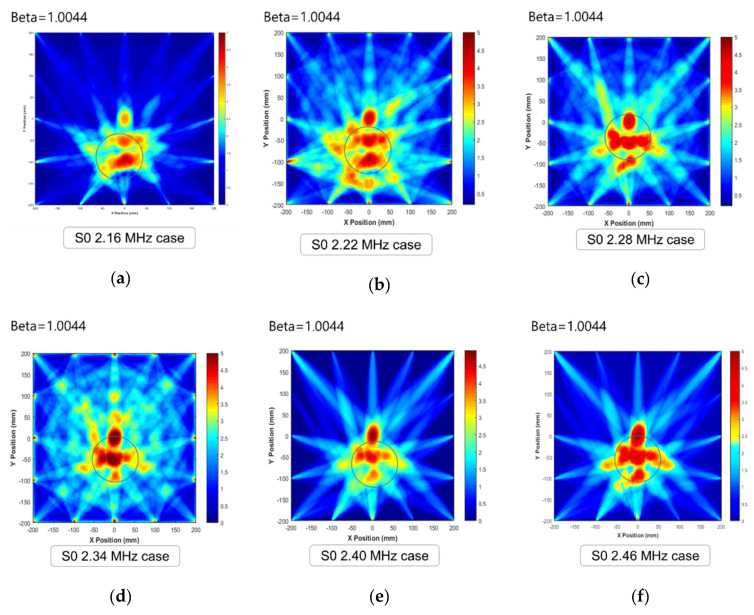
Tomographic images for S0 2.16 to 2.46 MHz·mm: (**a**) S0 2.16 MHz·mm, (**b**) S0 2.22 MHz·mm, (**c**) S0 2.28 MHz·mm, (**d**) S0 2.34 MHz·mm, (**e**) S0 2.40 MHz·mm, and (**f**) S0 2.46 MHz·mm.

To verify the serviceable mode of the Lamb wave among S0 2.16–2.46 MHz signals, visualization analysis was performed. At the beginning of the visualization process, considering the surroundings of the industrial field, the shape factor, calculated as the ratio of the defect size to the propagation distance, was set to 1.0044. The defect shape is shown in Figure 4a, and the tomographic images are shown in Figure 8. The results indicate that all the nondominant modes are able to indicate the location of the defect with a fixed shape factor (beta parameter). Figure 9 shows harsh illustrations from the viewpoint of high-resolution reconstruction. The primary reasons for roughness of the images are mode conversion effects and energy level distribution. The modes that do not indicate prominent wave intensity are not suitable for the quantification of images. In contrast, for the cases between 2.64 and 2.76 MHz symmetric modes, visualization images are indicated more clearly as compared to the other cases. This assessment is based chiefly on the dominance of wave mode and mode decomposition. Previous studies [4,5,6,7,8,9,10,11] have also strongly emphasized the importance of mode selection.

**Figure 10:**
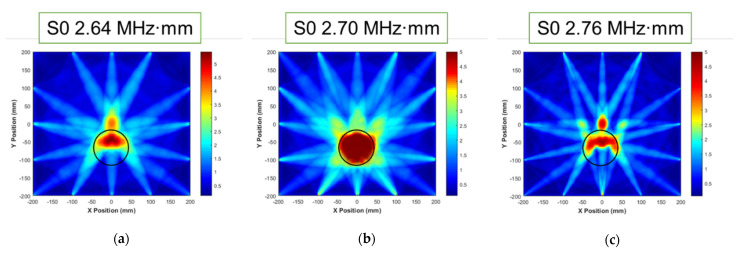
RAPID tomographic images for S0 2.64 to 2.76 MHz·mm: (**a**) S0 2.64 MHz·mm, (**b**) S0 2.70 MHz·mm, and (**c**) S0 2.76 MHz·mm.

For significantly dominant wave modes, the tomographic results were investigated for the same defect; the results are depicted in Figure 10. Compared with the previous results (shown in Figure 9), the quality of the images is enhanced. These results are used to revise shape accuracy and reliability. The case of S0 2.70 MHz·mm can be illustrated as a shape relatively similar to the detected shape. It was assumed that the suitable wave mode can be determined through a comparison with the other dominant modes.

To prevent distortion, the revised shape factor relied on the energy distribution pattern. The distortion effect was deduced using the beam pattern of the wave propagation, which depends on the energy distribution of the beam field. The remedy for a reliable tomographic method was revised based on analysis of the beam pattern and energy distribution with respect to the beam skewing effect.

### 4.3. Mode Verification of Antisymmetric Mode

**Figure 11:**
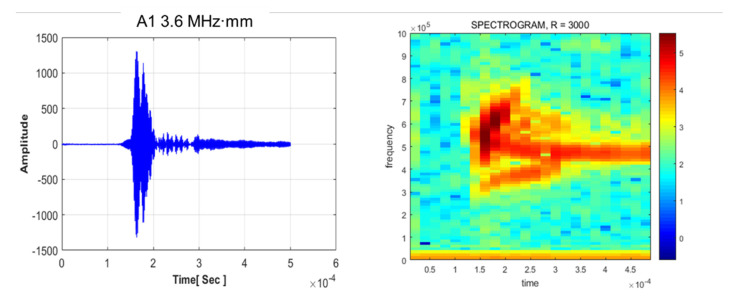
STFT analysis for antisymmetric Lamb wave mode.

**Figure 12:**
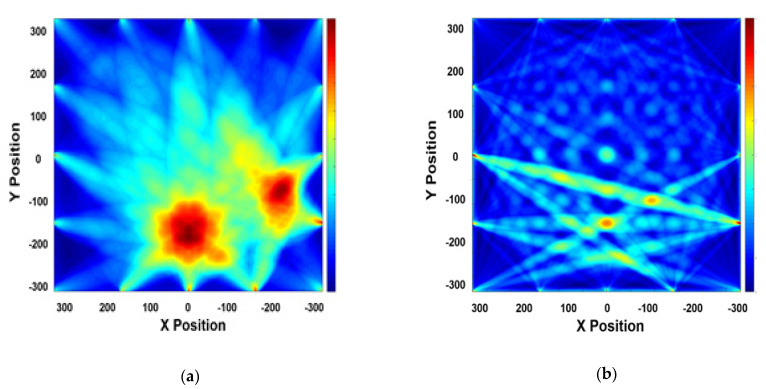
Comparison of RAPID tomographic images: (**a**) S0 3.0 MHz·mm and (**b**) A1 3.6 MHz·mm.

The antisymmetric mode presents a significant advantage in evaluating wall-thinning defects; it is affected by the transverse wave, which is related to shear stress. According to the theoretical background, the antisymmetric mode is more sensitive than the symmetric mode in evaluating wall-thinning failure. Thus, in this study, the reliability of the antisymmetric mode was confirmed, and STFT and tomographic tests were performed. The results of the STFT analysis are presented in Figure 11. Owing to the thickness of the plate, the A1 mode is not generated with dominance. Mode superposition effect is identified through the spectrogram and waveform shape. The ultimate goal of this research was the availability of visualization for defect cases. Referring to previous research [13], the multidefect case was applied to verify the quality of visualization. As shown in Figure 12, reconstruction of the image indicates trivial results compared with the S0 3.0 MHz·mm results. Therefore, the A1 mode was found to be unsuitable for the evaluation. This does not only demonstrate a superposition effect without dominance, but also yields insufficient tomographic results.

## 5. Shape Factor Modification through Beam Pattern Analysis

This section describes the methodology for correction of shape variables applicable to the field, targeting the mode S0 2.64–2.76 MHz·mm. The advantage of the RAPID algorithm is that it uses the shape of the wave lobe to correct the shape into an ellipse. Therefore, in this study, the shape function was corrected according to the size of the defect. Because the size and energy distribution of the main lobe differed according to the mode, the dynamics of the corresponding energy distribution were analyzed first; subsequently, the shape function application was studied. The underlying assumptions of the main lobe analysis method for applying the shape function were as follows:

- The main lobe, acoustic beam pattern, and propagation direction were determined by the guided ultrasonic mode.
- Based on the maximum energy level of the main lobe, lobe width was determined by assuming that the 50% energy level was the limit of the energy that can be thinned or defective.
- Based on this, a shape function was selected considering a lobe width of an elliptical shape; the difference between the defect image and the actual defect was analyzed by applying the shape function to the actual defect.

**Figure 13:**
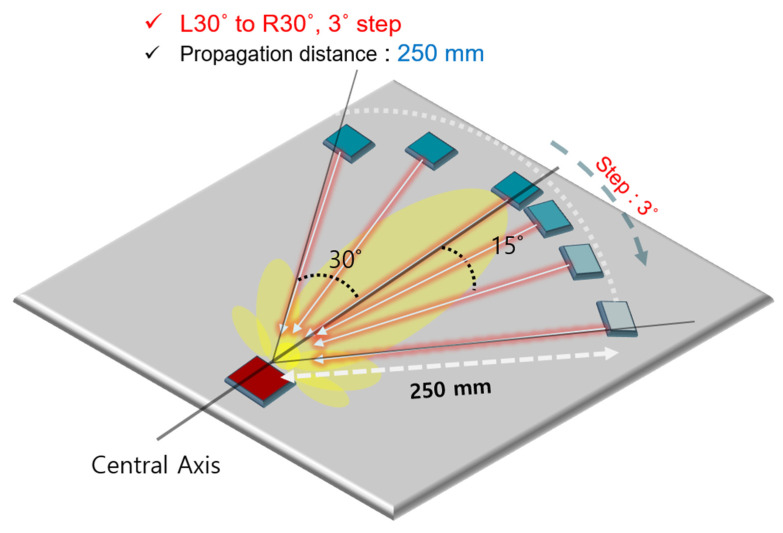
The concept of beam energy distribution and skewing analysis.

The beam width selection method is depicted in Figure 13. The energy level was measured at 3° intervals within 30° from the central axis. Measurements were conducted over a certain distance in the far-field section, but not in the near-field zone. The far-field zone was set to an area that was farther than two wavelengths from the sound source, and the group speed was about 1800 m/s. Therefore, it was regarded as far-field when the detection distance was 14 mm or longer at λ = 6.67 mm, for S0 2.70 MHz·mm. The measurements in this experiment were performed at a distance greater than 250 mm from the excitation source, which could be viewed as far-field.

**Figure 14:**
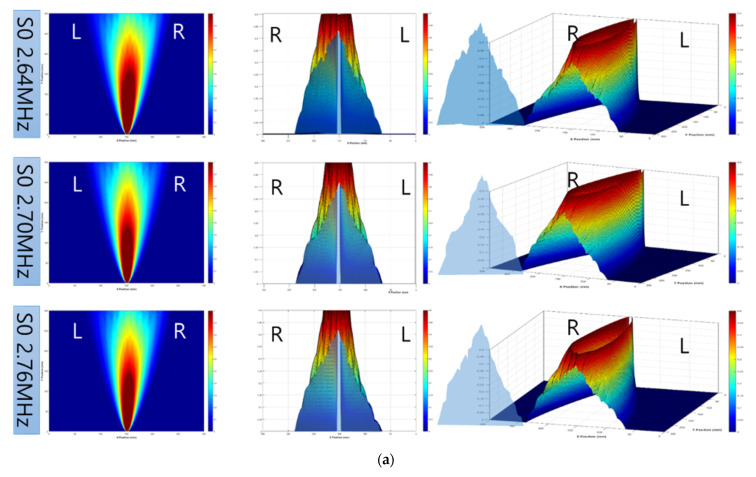

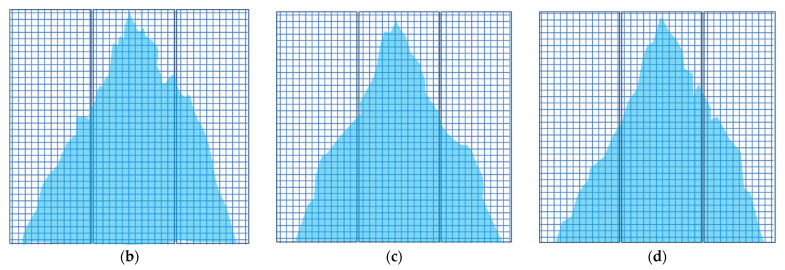
Energy distribution analysis for Lamb wave symmetric modes: (**a**) overall result, (**b**) S0 2.64 MHz·mm, (**c**) S0 2.70 MHz·mm, and (**d**) S0 2.76 MHz·mm.

Based on these conditions, the distribution of the energy levels for each mode was analyzed. The analysis results are presented in Figure 14; these results confirm that the left and right energy levels of the S0 2.70 MHz·mm mode are well balanced. It can be observed that the positional expression ability is high regardless of the position of the defect on the plane. In the case of 2.64 and 2.76 MHz·mm, the energy level is skewed to one side. Such a phenomenon may be determined by the conditions of the surface and the conditions within the specimen. It is possible that the S0 2.70 MHz·mm also exhibits a phenomenon similar to that shown by the other two modes. Considering the possibility of field application, a better correction method was required to improve the accuracy of the positional expression and defect distribution area.

**Figure 15:**
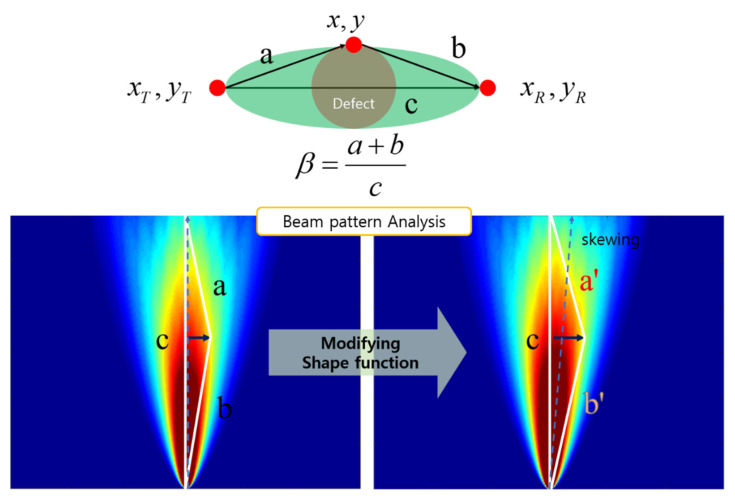
Proposed technique to determine shape variable β.

The revised method involved determining a shape function β according to the beam pattern, rather than determining a shape coefficient according to the defect. In the aforementioned method for applying RAPID imaging techniques, the inspected image was implemented by determining a shape function according to the defect shape. However, it was difficult to obtain images of inaccessible areas because the defect shape was not known before inspection. The greatest advantage of the guided ultrasound inspection technique was that it was possible to construct an inspection system based on a wide range of flaw detection areas and to image inaccessible or invisible areas. In areas where the defect was not visible, the defect image could not be determined at all, and even when the defect was implemented, the shape of the image was different from the original defect shape, as shown in Figure 15. Figure 15 illustrates the proposed method, wherein the original method of determining the shape function, introduced in Section 2 (refer to Figure 2), is modified.

**Figure 16:**
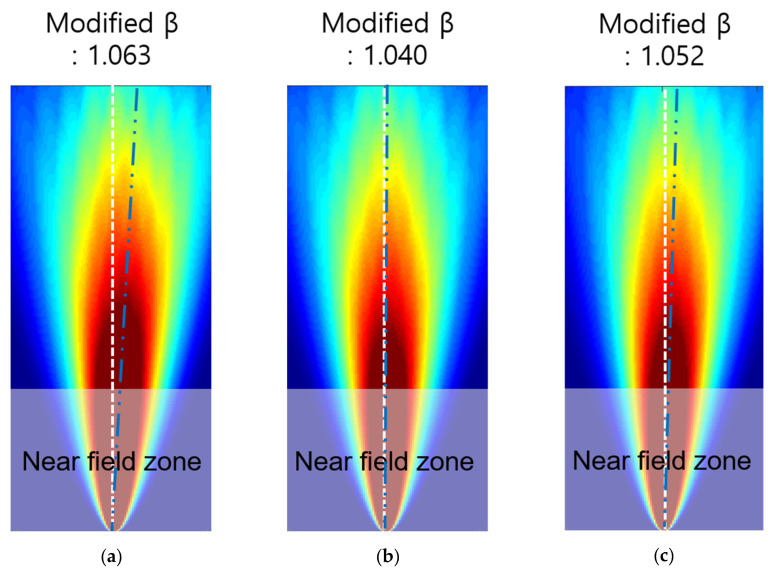
Revised shape function of variable *β* for RAPID tomography: (**a**) S0 2.64 MHz·mm, (**b**) S0 2.70 MHz·mm, and (**c**) S0 2.76 MHz·mm.

With the maximum range of the measurement area from the central axis of the plane to be tested, that is, the measurement flaw distance, the area corresponding to 50% of the maximum energy level of the skewed beam path was defined as the area where one beam width could be measured. The shape factor was selected accordingly based on the width formed. When the wave beam path was skewing, the path changed accordingly; therefore, the modified shape function was selected based on the distance from the central axis according to the modified path. The shape function values derived using this calculation method are shown in Figure 16.

**Figure 17:**
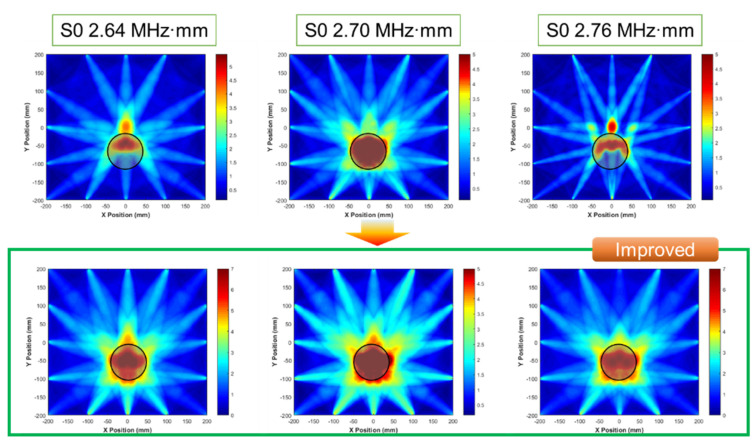
Modified tomography images using the proposed shape function approach by considering beam pattern analysis.

Using the modified shape function of the β parameter, the tomographic is reconstructed. Comparing with the previous results (as shown in Figure 10), improved tomographic images are illustrated as shown in Figure 17. The results show that the shape is very similar to the circular shape and distorted effects are supplemented. From the analysis of this modification effort, the resulting illustration is affected by beam skewing and beam diffraction effects.

In the case of field applications, the guided wave mode identification is needed to act on preprocessing, and a further step is to perceive the characteristic of the guided wave propagation pattern. Following the previous results, beam skewing and diffraction effects have to be clearly analyzed to reduce the distortion rate. In the case of harsh conditions to determine the cause of distortion effects, the proposed modification technique plays a significant role in the assessment. The technique is expected to remedy distorted effects and images more appropriately.

## 6. Conclusions

Following the application of the RAPID imaging process, the output of the modified RAPID technique was supplemented where the correct defect area was not visualized for some defects. Previous studies have attempted to increase the accuracy of the corresponding defects through the application of a variable shape factor, but this method has a limitation in that it is difficult to apply because of the environmental factors of the structure being imaged. To overcome this, an improved method for determining the shape function was studied, which was largely independent of the environmental constraints of the structure. For the 6 mm carbon-steel plate, the location possibility was determined through the application of the antisymmetric mode, and it was confirmed that quantitative imaging was very difficult in the bending stress dominant mode. The dominant mode was identified through frequency shifting and STFT, and it was confirmed that more accurate quantitative visualization of defects was achieved when imaging was performed through this mode. In addition, the imaging distortion caused by the beam skewing phenomenon, generated during the propagation of various guided ultrasound modes, could be corrected by applying a shape function modified through beam pattern and energy level analysis. Thus, a modified RAPID imaging algorithm was proposed, which determines the shape function considering the beam width and skewing rather than the defect shape.

The modified shape function was studied by the analysis of the propagating pattern for 6 mm plate cases. Further comparative research needs to verify another level of thickness. In addition, only circular shape defects were examined; nonetheless, this research was required prior to applying the method to various triangular, quadrature, and irregular shape defects. Fuzzy processing is also an alternative way to improve the quality of the image. From fuzzy process application, improved precision and accuracy of the image will be expected.

## Figures and Tables

**Table 1:** Group velocities of Lamb wave modes.

Wave Mode	Theoretical (m/s)	Experimental (m/s)	Error (%)
Rayleigh wave	2960	2980	0.7
S0 2.16 MHz·mm	2950	2850	3.4
S0 2.22 MHz·mm	2780	2830	1.8
S0 2.28 MHz·mm	2120	2120	0.1
S0 2.34 MHz·mm	2010	2090	4
S0 2.40 MHz·mm	1940	2000	3.1
S0 2.46 MHz·mm	1870	1920	2.7
S0 2.52 MHz·mm	1700	1830	4.1
**S0 2.64 MHz** **·mm**	**1710**	**1840**	**7.1**
**S0 2.70 MHz** **·mm**	**1850**	**1820**	**1.6**
**S0 2.76 MHz** **·mm**	**1910**	**1850**	**3.1**
A1 3.6 MHz·mm	2878	2930	1.8

## Data Availability

No new data were created or analyzed in this study. Data sharing is not applicable to this article.
